# Tetrazoles and Related Heterocycles as Promising Synthetic Antidiabetic Agents

**DOI:** 10.3390/ijms242417190

**Published:** 2023-12-06

**Authors:** Rostislav E. Trifonov, Vladimir A. Ostrovskii

**Affiliations:** 1 Department of Chemistry and Technology of Nitrogen-Containing Organic Compounds, Saint Petersburg State Institute of Technology (Technical University), St. Petersburg 190013, Russia; 2 Saint Petersburg Federal Research Center of the Russian Academy of Sciences (SPC RAS), St. Petersburg 199178, Russia

**Keywords:** tetrazoles, antidiabetic agents, type 2 diabetes mellitus, peroxisome proliferator-activated receptors (PPARs) agonists, aldose reductase (AR) inhibitors, dipeptidyl peptidase-4 (DPP-4) inhibitors, G protein-coupled receptor (GPCRs) agonists

## Abstract

Tetrazole heterocycle is a promising scaffold in drug design, and it is incorporated into active pharmaceutical ingredients of medications of various actions: hypotensives, diuretics, antihistamines, antibiotics, analgesics, and others. This heterocyclic system is metabolically stable and easily participates in various intermolecular interactions with different biological targets through hydrogen bonding, conjugation, or van der Waals forces. In the present review, a systematic analysis of the activity of tetrazole derivatives against type 2 diabetes mellitus (T2DM) has been performed. As it was shown, the tetrazolyl moiety is a key fragment of many antidiabetic agents with different activities, including the following: peroxisome proliferator-activated receptors (PPARs) agonists, protein tyrosine phosphatase 1B (PTP1B) inhibitors, aldose reductase (AR) inhibitors, dipeptidyl peptidase-4 (DPP-4) inhibitors and glucagon-like peptide 1 (GLP-1) agonists, G protein-coupled receptor (GPCRs) agonists, glycogen phosphorylases (GP) Inhibitors, α-glycosidase (AG) Inhibitors, sodium glucose co-transporter (SGLT) inhibitors, fructose-1,6-bisphosphatase (FBPase) inhibitors, IkB kinase ε (IKKε) and TANK binding kinase 1 (TBK1) inhibitors, and 11β-hydroxysteroid dehydrogenase type 1 (11β-HSD1). In many cases, the tetrazole-containing leader compounds markedly exceed the activity of medications already known and used in T2DM therapy, and some of them are undergoing clinical trials. In addition, tetrazole derivatives are very often used to act on diabetes-related targets or to treat post-diabetic disorders.

## 1. Introduction

Diabetes mellitus is an incurable disease defined by a metabolic disorder with hyperglycemia, contributing to a number of dangerous diseases: hypertension, thrombosis, neurodegenerative disorders, and so on. Hyperglycemia may be due to poor cellular susceptibility to insulin (insulin resistance) or type 2 diabetes mellitus (T2DM), as well as insufficient secretion of insulin by the pancreas (type 1 diabetes mellitus). Currently, the number of T2DM cases is constantly increasing, and the disease is now considered to be one of the non-transmittable chronic disease epidemics. The problem affects different populations, genders, and ages. Overall, T2DM accounts for about 90% of all cases of diabetes mellitus. To date, there are hundreds of millions of known documented cases of T2DM in the world. Note that not all cases of diabetes are reliably documented. According to International Diabetes Federation (IDF) data, the total number of people living with diabetes is projected to rise to 643 million by 2030 and 783 million by 2045 [1,2]. Type 1 diabetes mellitus can be controlled with the external administration of insulin. In contrast, a direct injection of insulin in the case of T2DM will not significantly reduce blood glucose. Here, drug therapy is required to target the various biological mechanisms responsible for metabolic processes. A great deal of studies have been dedicated to the development of such drugs, and these studies are being intensively developed [3].

**Figure 1:**
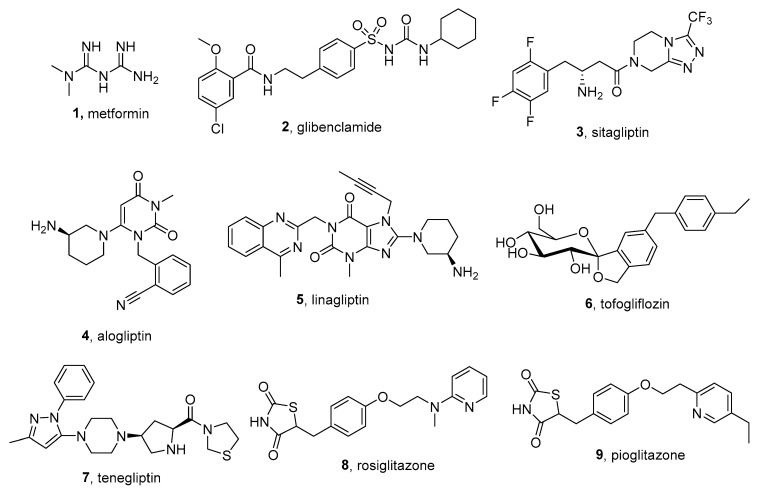
Some low-molecular oral antidiabetic medications: **1**—metformin (known since 1922, decreases glucose production in the liver, increases the insulin sensitivity of body tissues), **2**—glibenclamide (known since 1960s, stimulates insulin secretion by pancreatic β-cells, increases insulin release), **3**—sitagliptin (DPP-4 inhibitor, Merck), **4**—alogliptin (DPP-4 inhibitor, Takeda Pharmaceutical Company), **5**—linagliptin (DPP-4 inhibitor, Boehringer Ingelheim), **6**—tofogliflozin (SGLT-2 inhibitor, Chugai Pharma), **7**—teneligliptin (DPP-4 inhibitor, Mitsubishi Tanabe Pharma), **8**—rosiglitazone (PPAR agonist, GlaxoSmithKline), **9**—pioglitazone (PPAR agonist).

A large number of natural compounds with antihyperglycemic effects are known [4,5], but it is the synthetic drugs that are most widely used in the treatment of diabetes. Dozens of the biological targets of low-molecular antidiabetic agents are well recognized: the incretin hormones Glucagon-like Peptide-1 (GLP-1) and Glucose-Dependent Insulinotropic Polypeptide (GIP) themselves; their regulators such as Dipeptidyl Peptidase-4 (DPP-4); G protein-Coupled Receptors (GPCRs) such as GPR40, GPR120, and GPR119; Glucose Metabolism Pathway-Based Targets, such as Glucose Kinase (GK), Protein Kinase B (AKT/PKB); Insulin-Based Targets like Protein Tyrosine Phosphatase 1B (PTP1B); and other types of targets like Sodium-Glucose Cotransporter Protein-2 (SGLT-2), Peroxisome Proliferator-Activated Receptors (PPARs), etc. [6]. Antidiabetic drugs can have fundamentally different chemical structures, and they can hardly be assigned to a certain type. As can be seen, for example, from Figure 1, the following chemical compounds of different classes can be used as hypoglycemic agents: biguanides, sulfonylureas, thiazolidinediones, pyrimidines, purines, sugars, oligonucleotides, peptides, and many others [7,8]. In addition, other multiple medications are commonly used in combination with antidiabetic drugs to suppress secondary effects associated with T2DM and high blood sugar levels: cardiovascular and neurological agents, immunomodulators, and many others. Thus, medical treatments for T2DM are complex processes, and drug therapy, in combination with proper diet and lifestyle, can significantly improve the patient’s condition and reduce the dangerous consequences of hyperglycemia. Nevertheless, to date, there are no universal and highly effective drugs, either of natural or synthetic origin, to cure T2DM. The known drugs have a number of side effects, which has even led to a ban on the use of some of them [7]. Therefore, the development of more effective medications for the treatment of T2DM remains very important.

## 2. Tetrazoles for Biomedicine

It can be seen from Figure 1 and the cited literature sources that most of the known antidiabetic drugs in use include nitrogen heterocyclic moieties: azoles, azines, annelated azoloazines, oxazoles, thiazoles, and some others. The development of novel antidiabetic agents often involves the use of polynitrogen heterocyclic systems with more than two endocyclic heteroatoms (triazoles, tetrazoles, oxadiazoles, or their fused derivatives) as key scaffolds [8,9,10]. These moieties are very promising in drug design because they are often metabolically stable, easily participate in various intermolecular interactions through hydrogen bonding, conjugation, or van der Waals bonding with biological targets, and many of them are bioisosters of functional groups of endogenous molecules [11].

Among other heterocyclic systems, the tetrazole cycle has a unique structure and unique properties. Despite the fact that the tetrazole cycle contains only one endogenous carbon atom and four nitrogen atoms, it is a very thermally and metabolically stable system. Previously, the synthesis and properties of tetrazoles have been discussed in detail in numerous reviews and monographs, including by the authors of this review [12,13,14,15,16].

**Figure 2:**
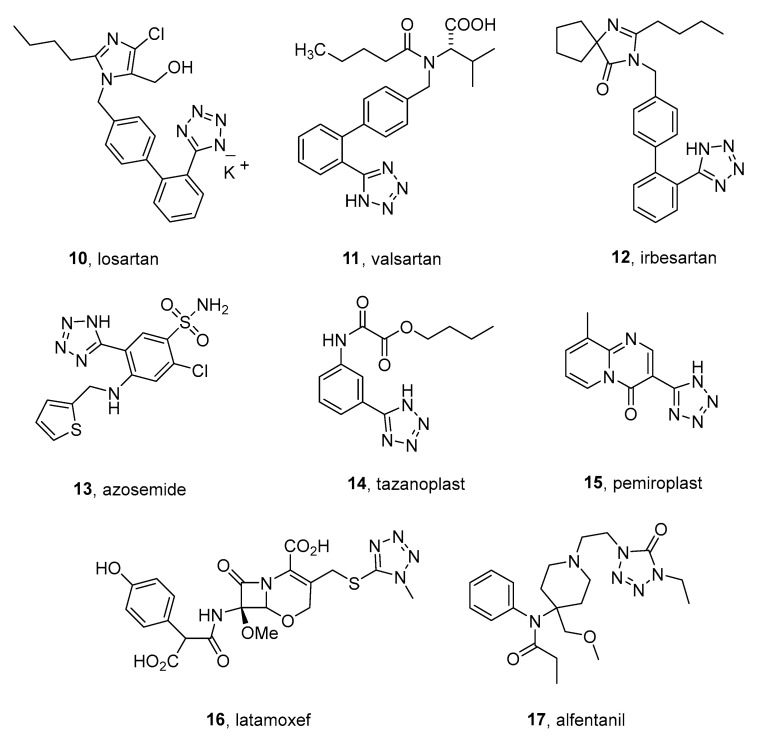
Some examples of tetrazole-containing medications.

Tetrazolyl moiety is present in the top 10 most frequent nitrogen heterocycles in US FDA-approved drugs and in a total of 16 pharmaceuticals [11]. This heterocycle is incorporated into active pharmaceutical ingredients of medications of various actions: hypotensives **10**–**12**, diuretic **13**, antihistamines **14**–**15**, antibiotics, analgesics, and some others (Figure 2) [17].

Quite a lot of research has been devoted to the medicinal chemistry of tetrazoles, the results of which are summarized in a number of reviews on this topic [16,17,18,19,20,21]. Significant achievements have been made in the field of drug development for hypotensive, anticancer drugs, semi-synthetic antibiotics, and agents acting on the central nervous system [17,19]. However, there are few data points, and there is no systematic analysis of the antidiabetic activity of tetrazole derivatives. In view of the above, this review can fill this gap.

**Figure 3:**
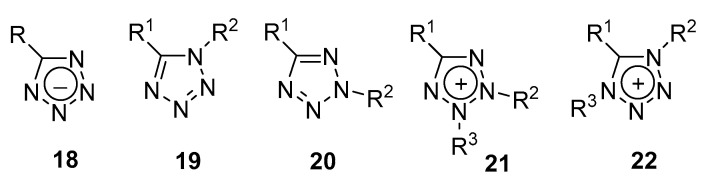
Main isomeric forms of tetrazoles.

Tetrazoles vary greatly in properties depending on their prototropic form and substituent isomerism (Figure 3).

Tetrazolate anions (tetrazolides) **18** are highly nucleophilic particles that are very soluble in water and other polar solvents and easily coordinate with transition metal ions [22]. Neutral 1*H*-tetrazoles **19** are usually more polar and thermally stable than 2*H*-tetrazoles **20**. Isomeric tetrazolium ions **21**, **22** are also very different in their properties. For example, cycle **21** can easily open under radiolysis, while ion **22** is still stable under these conditions [23]. The high stability of tetrazole forms can be partially explained with the sufficiently high aromaticity of the heterocycle. All the forms are planar and highly conjugated systems. Thus, the structural criteria of aromaticity for 2*H*-tetrazoles **20**, especially tetrazolides **18**, are close to those for benzene [24].

**Scheme 1:**
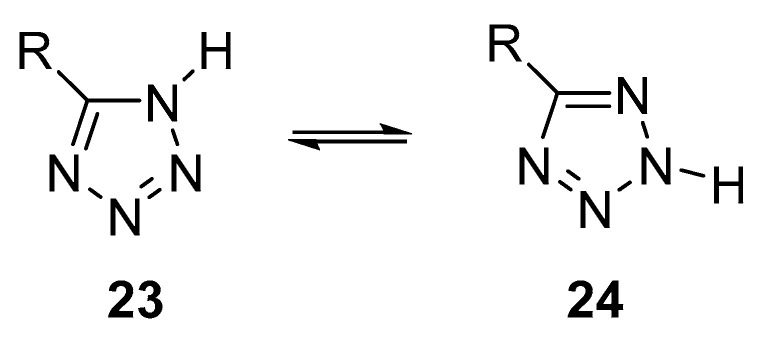
Tautomerism of NH-unsubstituted tetrazoles.

N*H*-Unsubstituted tetrazoles may exist as 1*H*- and 2*H*-tautomers **23** and **25** (Scheme 1). The most polar form **23** (where R is an electron-donor substituent or hydrogen) is preferred in condensed media (solutions or crystals), whereas the less polar 2*H*-form **24** with the same substituents prevails in the gas phase and non-polar solvents [25].

**Scheme 2:**
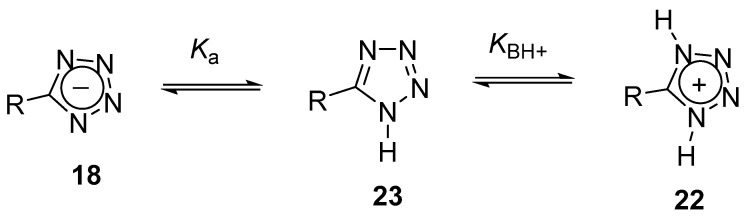
Ionizations of tetrazoles.

Tetrazoles are relatively strong N*H*-acids and weak bases (Scheme 2). The acidity of N*H*-tetrazoles **23** is comparable to that of aliphatic carboxylic acids. The acidity of parent tetrazole is p*K*_a_ 4.9, and depending on the nature of the *C*^5^-substituent, it can vary from 6 (for 5-aminotetrazole) to −1 (for 5-nitrotetrazole). Tetrazoles are also weak bases that ionize in strong mineral acids (for parent tetrazole, p*K*_BH+_ −2.7), and their basicity can vary in the range p*K*_BH+_ −1 ÷ −9 [25].

**Figure 4:**
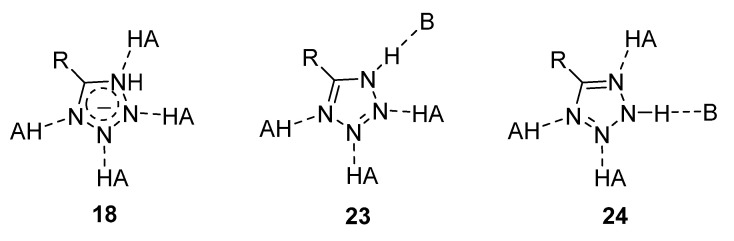
Possible ways of hydrogen bonding for different prototropic forms of 5-R-tetrazoles.

A special mention should be made of the unique ability of the tetrazole cycle to form stable hydrogen bonds simultaneously with several proton donors and acceptors (Figure 4). Such interactions are often predominant in the binding of active molecules to their biological targets. As we have recently shown, based on theoretical and experimental p*K*_HB_ values, tetrazoles are sufficiently strong bases for the formation of hydrogen bonding, and the substituent at position *5* of the cycle has a noticeable effect on the basicity [26].

It is now generally accepted that neutral 1*H*-tetrazole forms **19**, **23** are metabolically stable bioisosteric analogs of *cis*-amide and carboxyl groups, and tetrazolide **18** is an analog of carboxylate anion. However, in recent years, this concept of bioisosterism has been refined. As shown by Allen and co-authors, based on the analysis of crystallographic data and the results of theoretical calculations, the nature of hydrogen bond formation in pairs—1*H*-tetrazole-COOH and tetrazolide-carboxylate—is somewhat different [27]. The authors of the cited work also indicated that the functional groups of biomolecules linked by hydrogen bonds to 1*H*-tetrazole or tetrazolate anion are located at a distance greater by approximately 1.2 Å compared to the isosteric fragments of -COOH and -COO^−^. Our recent quantitative studies of the hydrogen bonding basicity of tetrazoles also support the fact that their ability to form hydrogen bonds is unique and significantly different from the carboxylic group [26].

Further, we have tried to analyze and structuralize the known studies, which examined the direct antidiabetic action of tetrazole derivatives towards known established biological targets. The sequence of the materials is presented in accordance with the effectiveness and scope of the results obtained for bioactive tetrazoles, and it is structured according to the targets of action.

We intentionally omit a large number of publications in which biologically active tetrazoles are considered components of the complex therapy of T2DM. For example, sartans **10**–**12** and angiotensin II receptor (ATR) blockers are very often used in this context, and ATRs are considered diabetes-associated targets. The use of sartans prevents the development of diabetes in hypertensive patients and reduces the progression of cardiovascular and renal diseases [28].

## 3. Low-Molecular Antidiabetic Agents Containing Tetrazolyl Moiety

### 3.1. Peroxisome Proliferator-Activated Receptors (PPARs) Agonists

**Figure 5:**
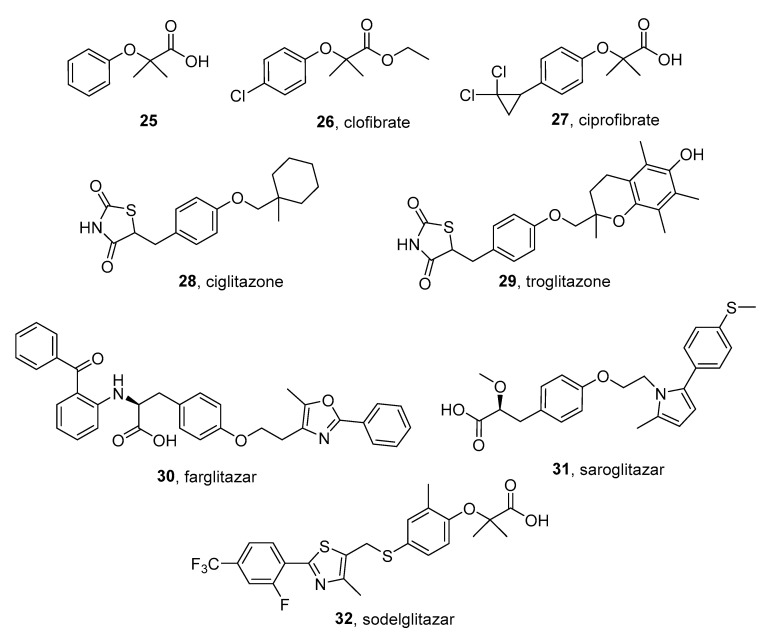
Some low-molecular PPAR agonists: fibric acid **25** and its derivatives **26**, **27** (PPAR-α); thiazolidinediones **28**, **29** (PPAR-γ); glitazars **30** (PPAR-γ), **31** (dual PPAR-α and PPAR-γ), **32** (all PPARs).

Peroxisome proliferator-activated receptors (PPARs) belong to a large family of ligand-inducible nuclear hormone transcription factors that regulate the expression of genes responsible for the differentiation and function of adipose tissue, lipid metabolism, the severity of the inflammatory response, and the production of cytokines and adhesion factors by cells. Three isoforms have been identified in human PPAR: PPARα, PPARγ, and PPARβ/δ [29]. Each isoform is encoded by its specific gene and has its own tissue specificity. PPARα is expressed mainly in liver, brown fat, kidney, heart, and skeletal muscle cells; PPARγ is expressed in adipose tissue and small intestine; and PPARβ/δ is expressed in skeletal muscle, heart, adipose tissue, and keratinocytes [30]. PPARα exerts control in lipoprotein assembly and also influences the rate of cholesterol synthesis in hepatocytes. Two isoforms—PPARγ1 and PPARγ2 are known. The PPARγ2 isoform is found in adipocytes, whereas PPARγ1 is common in skeletal muscle. PPARγ regulates tissue glucose content through changes in insulin receptor sensitivity. The insulin-sensitizing effect of this receptor isotype has been shown to be based on stimulation of proliferation of white adipose cells, which are highly sensitive to insulin. PPARδ/β is expressed in many tissues and acts as a sensor of polyunsaturated fatty acids. Its overexpression enhances fatty acid oxidation in mitochondria and also reduces obesity and insulin resistance. PPARs are short-lived proteins whose activity changes rapidly in response to ligands (agonists). Such ligands can be fatty acids, endogenous fatty acid metabolites, or synthetic substances. It should be noted that the development of synthetic drugs acting on PPARs is very promising due to the fact that these proteins are highly conserved [29]. In principle, action on all three PPAR isotypes can be successfully applied in the therapy of T2DM, metabolic syndrome, and related diseases. Synthetic chemical compounds that act as agonists of one, two (PPARα, PPARγ), or all isotypes of these receptors are known (Figure 5) [30,31]. Nevertheless, today, the major part of the research in drug design for the treatment of T2DM involves the consideration of PPARγ as a target [8]. It should be noted that some representatives of these ligands, such as thiazoldienones, exhibit significant toxicity [30]. The development of new agonists of PPARs, especially PPARγ agonists, has been a very urgent task for a long time.

**Figure 6:**
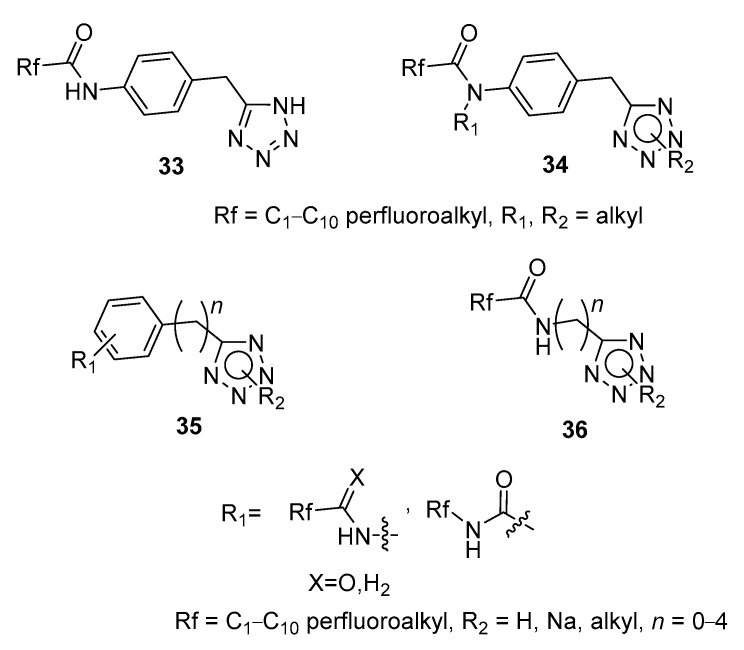
Perfluoroalkyl tetrazoles as antihyperglycemic agents.

Kees et al., in 1989, synthesized and investigated in vivo the hypoglycemic activity of a number of tetrazol-5-yl-perfluoro-anilides **33**,**34** on two genetic animal models: obese (ob/ob) and diabetic (db/db) mice (Figure 6). According to the results obtained, perfluorooctanoyl and perfluorononanoyl derivatives showed greater efficacy in normalizing glucose and insulin levels than ciglitazone **28** [32]. Later, these authors somewhat extended the discussed compounds by considering not only perfluoronanilides but also related perfluorocarbon derivatives **35**,**36**, their salts, and so on [33,34]. Whereas the thiazolidinediones require the acidic proton on the heterocycle for activity, tetrazolyl derivatives of perfluoro anilides retained antihyperglycemic efficacy in ob/ob mice after alkylation. Although the authors did not specify the biological target for these compounds, tetrazoles **33**–**36** can be considered structural analogs of typical PPARγ inhibitors-thiazolidinediones. Significant side effects were also observed for these compounds.

**Figure 7:**
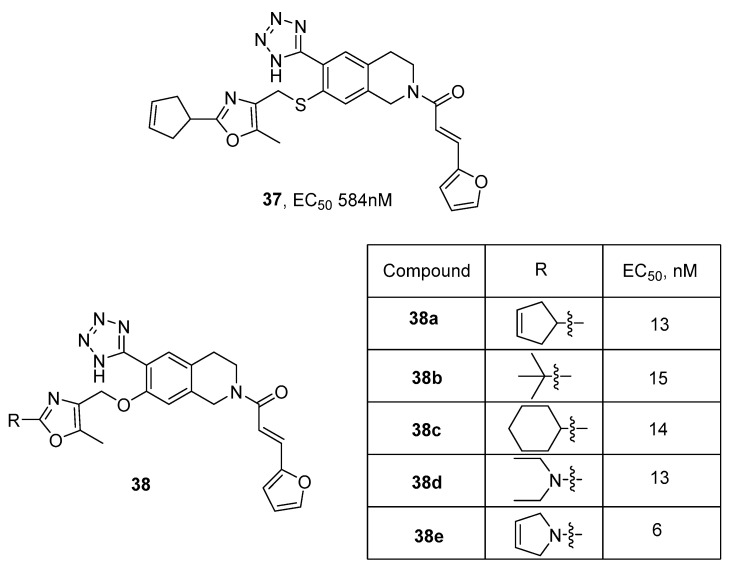
Tetrazol-5-yl-1,2,3,4-tetrahydroisoquinolines as selective PPAR-γ agonists.

It is known that among the derivatives of 1,2,3,4-tetrahydroisoquinoline-3-carboxylic acid, effective compounds that are to be PPAR agonists with protein tyrosine phosphatase 1B (PTP1B) inhibition have been found [35]. Morishita et al., in 2019, first synthesized two tetrazolyl derivatives **37**, **38a** based on the related 1,2,3,4-tetrahydroisoquinoline scaffold (Figure 7) [36]. One of these compounds, **38a**, proved to be ten times more active than the well-known drug farglitazar **30** (10^−7^ M) and selective PPARγ agonist in vitro on COS-1 cells with the human RXRα plasmid and electroporated full-length human PPAR plasmids. At the same time, the effect of **38a** on other PPARs and PTP1B was insignificant. The substitution of an oxygen atom for a sulfur atom in the linker fragment strongly reduced the activity (Figure 7). The significant affinity of **38a** for PPARγ was also confirmed with theoretical calculations and the interaction of the tetrazolyl fragment with amino acid residues of the protein occurring via salt bridges and hydrogen bonds. Other heterocycles did not show such high activity in this case. Later, the authors extended the series of seven-substituted-2-[(*E*)-3-(2-furyl)-acryloyl]-6-tetrazolyl-1,2,3,4-tetrahydroisoquinoline derivatives **38a**–**e** that were synthesized and their PPARγ partial agonist activity in vitro on COS-1 cells was studied [37]. In this series, a number of compounds turned out to be very active, and the leader was a derivative containing a dihydropyrrole moiety **38e**, which was marked by the authors as KY-903 (Figure 7). The PPARγ agonist potency of **38e**, based on its EC_50_ value, was 68-fold stronger than that of pioglitazone **9** [38]. It was once again pointed out that the replacement of tetrazolyl fragments, even with a structure-closed substituent, dramatically affected the activity of the compound. In vivo studies of the compound-leader KY-903 **38e** effects on KK-Ay mice and OVX/HFD rats showed that this compound has weaker adverse effects than pioglitazone **9**, such as increases in body and adipose tissue weights and bone loss are attributed to adipogenesis induced by PPARγ activation [38].

**Figure 8:**
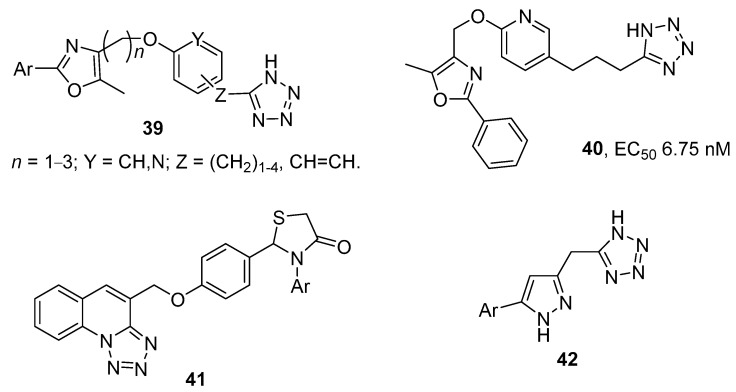
Tetrazole analogs of glitazones as PPARγ agonists.

A wide series of five-substituted N*H*-tetrazoles, most of which can be described with the general formula **39**, as potential PPARγ agonists were synthesized by Momose et al. (Figure 8) [39]. Their activity as PPARγ agonists in vitro on PPARγ:RXRα:PPRE34/CHO K1 cells and lipid-lowering activities in vivo on KKAy mice and Wistar fatty rats were studied. These compounds may be considered as glitazole analogs where thiazolidinedione moiety is substituted with the tetrazolyl one. Leader compound, 5-[3-[6-(5-methyl-2-phenyl-4-oxazolylmethoxy)-3-pyridyl]propyl]-1*H*-tetrazole **40**, possessed high antidiabetic effects due to its potent agonistic activity for PPARγ (EC_50_ 6.75 nM) and potent glucose-lowering activity (ED_25_ 0.0839 mg kg^−1^day^−1^), being 72 times more active than pioglitazone hydrochloride **9** (ED_25_ 6.0 mg kg^−1^day^−1^).

A series of three-substituted phenyl-2-(4-(tetrazolo[1,5-a]quinolin-4-ylmethoxy)phenyl)thiazolidin-4-ones **41** were synthesized, and the effects of these compounds on the improvement of oral glucose tolerance in a sucrose-loaded rat model were investigated (Figure 8) [40]. Thiazolidinones **41** displayed notable in vivo antihyperglycemic activity compared with metformin **1**, and it was proposed that they interact with active sites of PPARγ. One more analog of glitazones-5-(pyrazol-3-yl)-1*H*-tetrazoles **42** was obtained (Figure 8) [41]. Their agonistic property via PPAR-trans-activation assay was studied but none of them demonstrated any significant activity. According to results of in vivo antihyperglycemic activity studies of compounds **42** in sucrose loaded models on Sprague–Dawley rats, only one compound (Ar = 4-CH_3_S(CH_2_)_2_C_6_H_4_) demonstrated 24.6% of blood glucose lowering activity, which is two times higher than that of metformin **1**.

**Figure 9:**
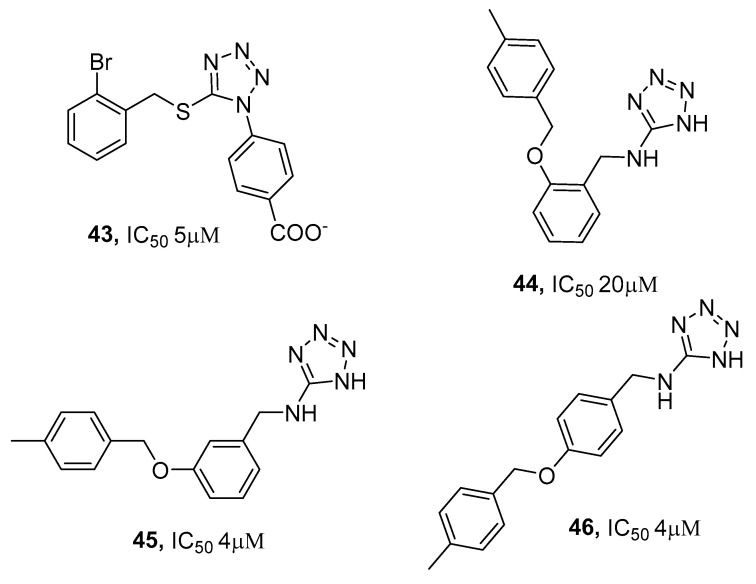
Tetrazole analogs of thiazolidinones as PPAR-γ agonists.

From the data of the paper [42], the computational analysis suggested that mercapto tetrazole **43** will bind to PPARγ selectively (Figure 9). However, according to the in vitro experiment against this receptor, its activity was lower than for rosiglitazone **8**. In this experiment, hPPARγ-LBD was cloned into pET28a plasmid and transformed in BL21 DE3 *Escherichia coli* strain. A little later, the same authors investigated three isomeric tetrazole derivatives **44**–**46**, structurally similar to thiazoldienones, using theoretical and experimental methods (Figure 9) [43]. According to the results obtained, all isomers are able to bind effectively to the PPARγ ligand binding domain, but the binding energy is still somewhat lower than for rosiglitazone **8**. This is in agreement with the experimental data in vitro determined in this assay: rosiglitazone **8** IC_50_ 160 nM, **44** IC_50_ 20 µM, **45** and **46** IC_50_ 4 µM. The crystal structure of the PPARγ/**45** complex was determined, confirming the binding mode.

**Figure 10:**
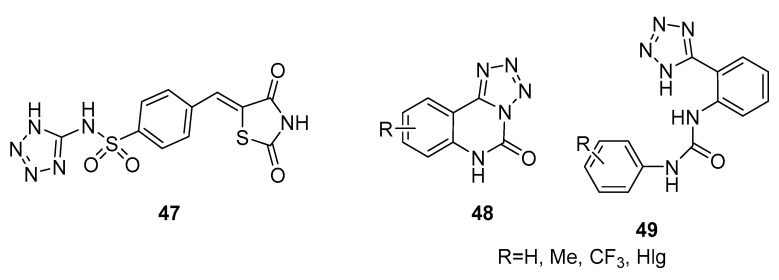
Tetrazolyl sulfamides, ureas and biguanides as PPAR-γ agonists.

Pattana et al. synthesized sulfamide **47** containing tetrazol-5-yl moiety (Figure 10), and most of them showed good activity compared to glibenclamide **2** [44]. Two series of tetrazolyl derivatives as possible hypoglycemic agents (tetrazolo[1,5-c]quinazolin-5(6*H*)-ones **48** and 1-[2-(1*H*-tetrazol-5-yl)aryl]-3-aryl(ethyl)ureas **49**), belonging to ureas or biguanides antidiabetic agent types, were synthesized by Antypenko et al. (Figure 10) [45]. According to the molecular docking calculations, compound **49** (R_1_ = H, R_2_ = 4-CF_3_) demonstrates a good affinity to the PPARγ receptor with −10.1 kcal mol^−1^. Dexamethasone diabetes models (glucose tolerance, oral rapid insulin, and adrenalin tests) were used to determine the specific hypoglycemic activity, and compound **48** (R=H) revealed high activity, exceeding the reference drugs metformin **1** and gliclazide on White Wistar rats.

**Scheme 3:**
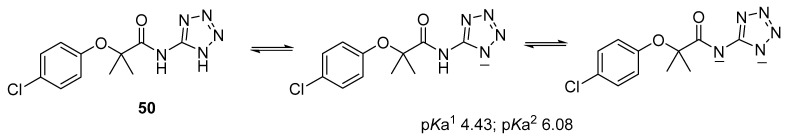
Dissociation of tetrazole analog of clofibric acid.

**Scheme 4:**
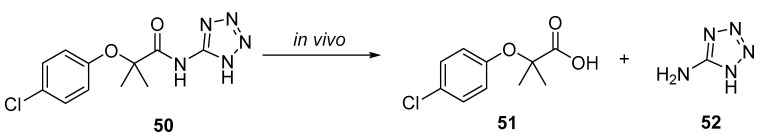
Hydrolysis of tetrazole analog of clofibric acid in vivo.

The tetrazole analog of clofibric acid **50**-2-(4-chlorophenoxy)-2-methyl-N-(1*H*-tetrazol-5-yl) propanamide, presumed to be a PPARα agonist, was obtained, and its properties were studied [46,47]. The antidiabetic activity of compound **50** was determined at 50 mg kg^−1^ single dose using the diabetes mellitus rat model. The results indicated a significant decrease in plasma glucose levels during the 7 h post-administration. This compound may exist in dissociated form (Scheme 3) and should be metabolized in vivo with the release of free clofibric acid **51** (Scheme 4). At the same time, the overall toxicity of tetrazole **50** is lower than that of clofibric acid **51**. Thus, compound **50** may be considered a prodrug of **51** as an antidiabetic and anti-dyslipidemic agent.

### 3.2. Protein Tyrosine Phosphatase 1B (PTP1B) Inhibitors

**Figure 11:**
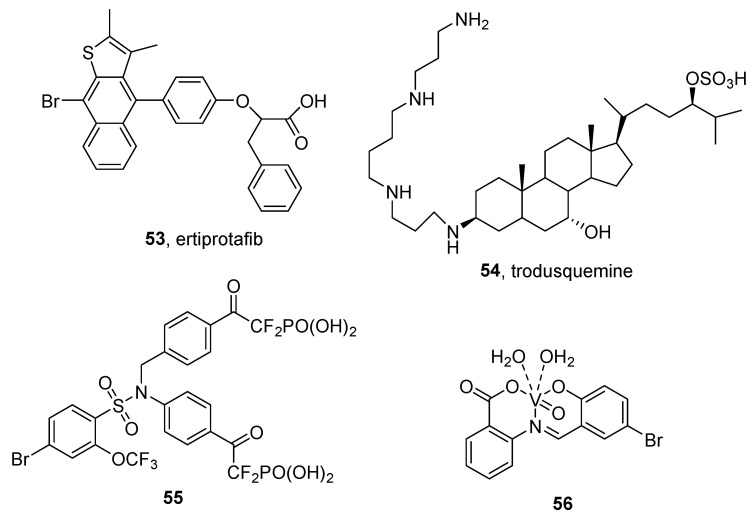
Low-molecular PTB1B inhibitors.

The insulin and leptin action is controlled through a balance between the phosphorylation and dephosphorylation of insulin and leptin receptors. The action of insulin and leptin is co-directed and results in increasing energy stores in glycogen form in muscle and liver as triglycerides in adipose tissue. The intracellular enzyme protein tyrosine phosphatase 1B (PTP1B) is a negative regulator of the insulin and leptin signaling cascades: its activation leads to the sensitivity-lowering of the corresponding receptors [48,49]. PTP1B can also influence other biological processes and is expressed in multiple tissues, including the skeletal muscle, liver, adipose tissue, and brain [50]. PTP1B inhibitors enhance insulin and leptin signaling and could potentially improve insulin resistance, normalizing plasma glucose and insulin levels without inducing hypoglycemia [51]. Recently, various PTP1B inhibitors have been developed as drug candidates for T2DM therapy (Figure 11) [8,48,51]. These compounds belong to various types of organic or inorganic compounds: aromatics, nitrogen-containing heterocycles, sulfamides, semi-synthetic terpenes, oligonucleotides, ureas, and vanadium complexes [48,52]. Thiazoldienones and some other typical agonists of PPARs can also act as inhibitors of PTP1B [8]. Thus, a number of the tetrazole PPAR ligands discussed above also weakly inhibit PTP1B; for example, compound **38a** IC_50_ 1100 nM [36].

Based on docking and MD modeling, Murthy and Kulkarni suggested that compounds containing tetrazolyl functional groups might exhibit relatively good activity as PTP1B inhibitors. Also, it is possible to incorporate sulfonate or tetrazolyl moieties instead of phosphate ones in the further design of compound-leaders. These results are supported using surface area calculations and are consistent with the inhibitor activity of the compounds [53].

**Figure 12:**
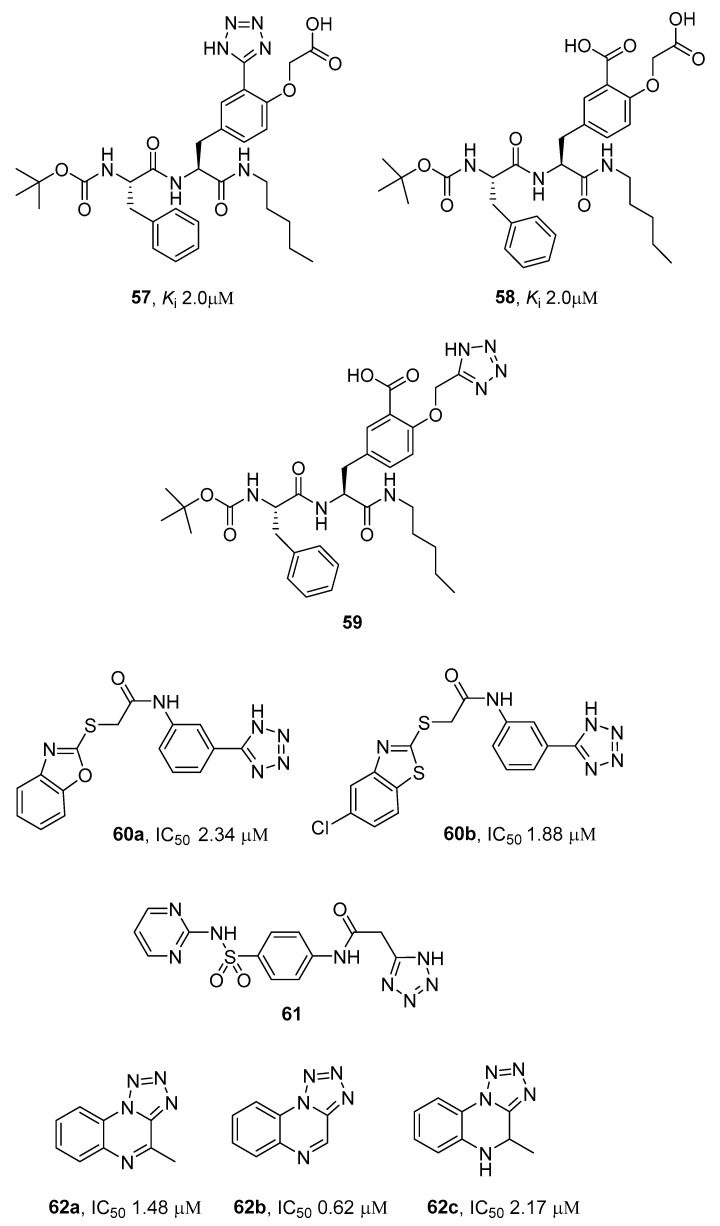
Low-molecular tetrazoles as PTB1B inhibitors.

Small molecular-weight peptidomimetics as competitive inhibitors of PTP1B have been synthesized by Liljebris et al. (Figure 12) [54]. Tetrazole **57** showed good PTP1B inhibitory activity (*K*i 2.0 µM), which was equipotent to the dicarboxylic acid analog **58**. The X-ray cocrystal structure of **57** and PTP1B revealed that the tetrazolyl moiety is positioned in the active site and binds similarly to carboxylate analog **58**. It should be noted that isomeric compound **59** did not show such activity.

A series of non-carboxylic inhibitors of PTB1B-N-(3-(1*H*-tetrazole-5-yl) phenyl) acetamides was synthesized and evaluated in vitro (Figure 12) [55,56]. Two compounds **60a**,**b** were found to exhibit good inhibitory activity against PTP1B. The active compounds showed the requisite binding interactions with amino acid side chains in the catalytic site of PTP1B, as indicated in molecular docking studies [55]. The leader compound **60b** also showed comparable antidiabetic efficacy to that of standard antidiabetic drugs in two in vivo models [56]. An example of benzene-sulfonamide tetrazole **61** was synthesized using sulfadiazine as a starting material (Figure 12) [57]. The molecular modeling study showed the compounds’ high affinity and selectivity to the active site and B-site of PTP1B, holding hydrogen bonding, hydrophobic, and electrostatic interactions. Based on computational design, the structure of terazoles **62a**–**c** as PTP1B inhibitors was proposed. In this case, a good concordance with in vitro results on recombinant 322 amino acids PTP1B was observed (Figure 12) [58].

### 3.3. Aldose Reductase (AR) Inhibitors

**Figure 13:**
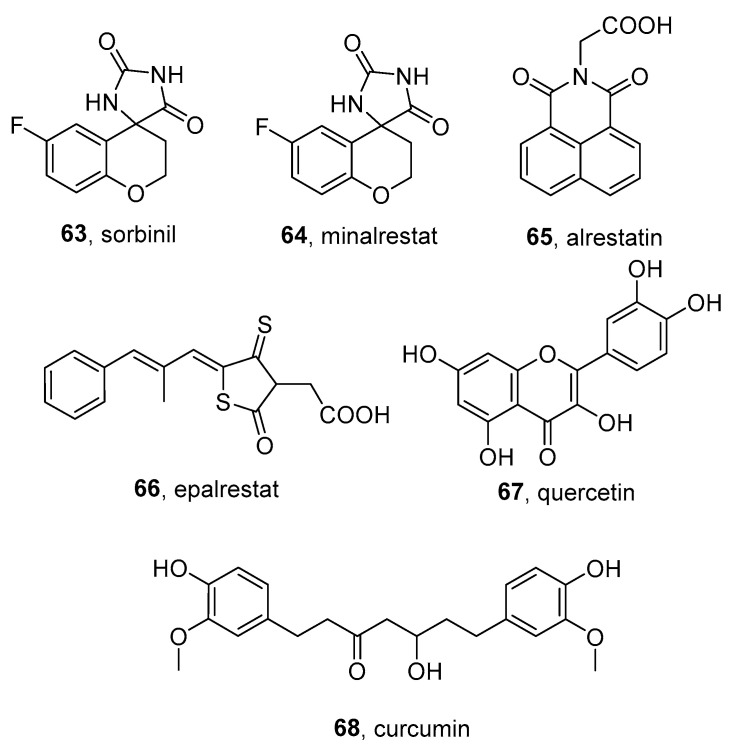
Well-known low-molecular AR inhibitors.

Aldose reductase (AR) is the first enzyme of the so-called polyol pathway, which converts glucose to sorbitol. It forms part of the aldo-keto reductase superfamily and is classified as AKR1B1. Sorbitol is subsequently converted to fructose in the presence of sorbitol dehydrogenase [8,59]. In hyperglycemic conditions with poor sorbitol penetration and metabolism, an excessive accumulation of sorbitol occurs in tissues, resulting in T2DM-associated complications such as cataracts, glaucoma, nephropathy, and vascular complications. Thus, normally, only less than 3% of glucose is converted to sorbitol, whereas in the case of DM, this amount is about 30%. Hence, inhibition of AKR1B1 has been seen as a plausible therapy for preventing complications arising from T2DM. The development of low-molecular AKR1B1 inhibitors has a rich history and has been ongoing for more than 50 years. To date, dozens of such compounds are known. Most aldose reductase inhibitors can basically be divided into three main classes: cyclic imides **63**,**64**, carboxylic acids **65**,**66**, and polyphenols **67**,**68** (Figure 13) [59].

**Figure 14:**
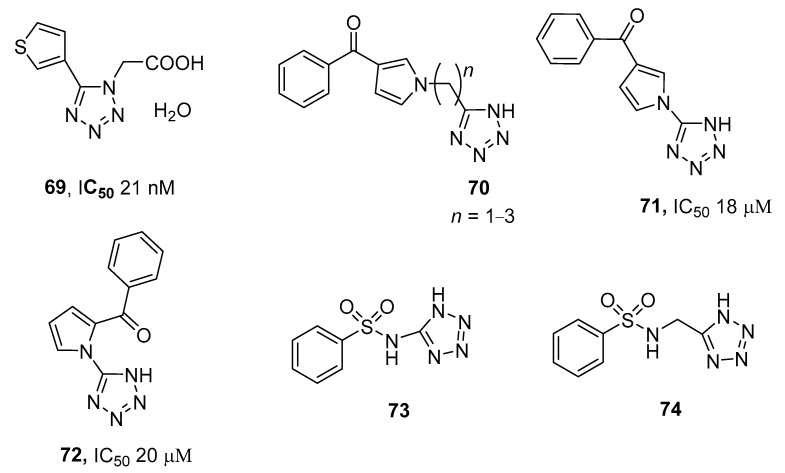
Tetrazoles as AKR1B1 inhibitors.

The first example of a tetrazole derivative as an aldose reductase inhibitor was made by Inukai et al. in 1993 [60]. The [5-(3-thienyl)tetrazol-1-yl]acetic acid **69** (Figure 14) demonstrated high inhibition of partially purified AKR1B1 from rat lens (IC_50_ 21 nM), rabbit lens (IC_50_ 23 nM), and human placenta (IC_50_ 28 nM). The oral administration of **69** (5–100 mg kg^−1^ day^−1^) to (STZ)-induced diabetic rats during a 5-day treatment period decreased the sorbitol content in the sciatic nerve, dose-dependently (ED_50_ 8.8 mg kg^−1^ day^−1^ for the prevention and 9.0 mg kg^−1^ day^−1^ for the reversal). A little later, Hotta et al. investigated the in vivo properties of tetrazole **69** in more detail. Physiological and biochemical studies were subsequently conducted on rat nerve tissue, and erythrocyte sorbitol content was estimated. Sciatic nerve blood flow was markedly lower (about 43.4%) in untreated diabetic rats than in non-diabetic controls [61]. The results obtained suggest that compound **69** has therapeutic value for diabetic retinopathy [62,63,64].

Another type of tetrazolyl-containing aldose reductase inhibitors was obtained and studied by Nicolaou et al. [65]. The pyrrolyl-tetrazoles **70**–**72** have significantly lower activity in vitro, regardless of isomerism and linker chain length (Figure 14). Weakly active were also found to be phenylsulfonamide derivatives **73**,**74**: 45% and 24% inhibition at 100 µM, respectively [66].

### 3.4. Dipeptidyl Peptidase-4 (DPP-4) Inhibitors and Glucagon-like Peptide 1 (GLP-1) Agonists

Incretin hormones—glucagon-like peptide 1 (GLP-1) and glucose-dependent insulinotropic polypeptide (GIP)—have a direct influence on glucose homeostasis. They are secreted by L- and K-cells in the intestinal mucosa in response to nutrient intake. These hormones are responsible for the most amount (up to 70%) of insulin secretion whilst suppressing glucagon secretion. However, GLP-1 and GIP are quite unstable, and under the action of enzyme DPP-4, they rapidly cleave within several minutes [8,67]. In type 2 diabetes, the function of incretins is reduced, and insulin secretion is impaired. It should be noted that GLP-1-dependent stimulation of insulin secretion occurs only under conditions of hyperglycemia, and the risk of hypoglycemia is very low. Thus, by inhibiting the action of DPP-4, it is possible to maintain the necessary amount of incretins in the organism. It is now reliably established that DPP-4 is a very convenient target for the action of oral antidiabetic agents, and research on the development of its inhibitors has been very intensively developed in the last two decades. Significant results have been achieved here for the treatment of T2DM. The structures of some known DPP-4 inhibitors, so-called glyptins **3**, **4**, **5**, **7**, are shown in Figure 1. More effective compounds are now being developed. It can be noted that practically all such compounds contain polynitrogen heterocyclic moieties [9]. Given this, one would expect that tetrazoles would also be in demand in this context, but there are few such publications.

**Figure 15:**
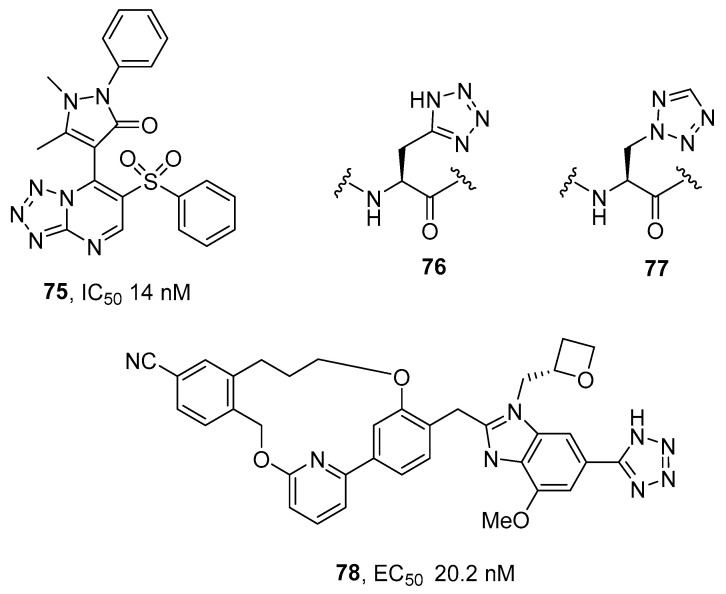
Tetrazoles as DPP-4 inhibitors and GLP-1 agonists.

In the work cited earlier, Antypenko et al., by means of molecular docking calculations, showed a good affinity of **48** and **49** to DPP4 [45]. Gomha et al. synthesized tetrazolo[1,5-a]pyrimidine **75** (Figure 15), which exhibited high activity in vitro examined on DPP4 with the fluorescent method (IC_50_ 14 nM) [68]. The assays in mice demonstrated that the administration of compound **75** at a dose of 200 mg kg^−1^ for 7 days led to a decrease in serum glucose by 41.3%, comparable with sitagliptin (43%).

Acting essentially within the same biological process of the glucose-dependent stimulation of insulin secretion while suppressing glucagon secretion, it is possible to activate the action of GLP-1 through interaction with appropriate ligands-peptides (exendin-4, liraglutide) or some small molecules. Thus, in the UK patent, the preparation and activity as GLP-1 receptor agonists of a series of peptides, containing tetrazol-5-yl and tetrazol-2-yl moieties **76**, **77**, have been proposed (Figure 15) [69]. Some of these peptides have demonstrated high activity in vitro and in vivo. A synthesis and quite significant biological activity in vitro on HEK293 cell line with 581 fmol mg^−1^ of GLP-1R of the macrocyclic agonist of GLP-1 **78** was also described in the WO patent of Eli Lilly Company [70].

### 3.5. G Protein-Coupled Receptor (GPCR) Agonists

**Figure 16:**
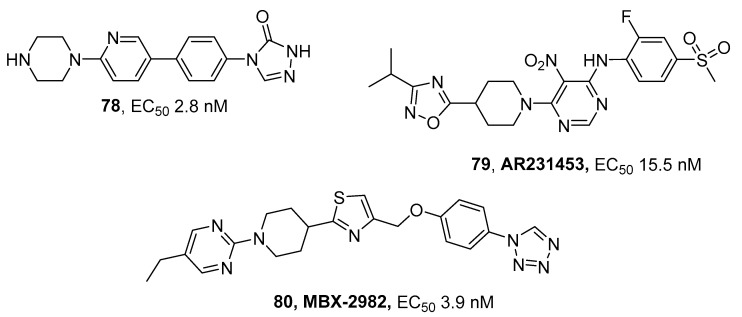
Polynitrogen heterocyclic compounds known as GPR119 agonists.

Another way that influences the production of incretin hormones GLP-1 and GIP is through the activation of some G protein-coupled receptors (GPCRs) known as cell surface receptors. These receptors mediate internal signal transduction via the detection of molecules on the cell surface, and therefore, they can be activated by low-molecular biological active compounds [8,71]. GPR119, mainly, but also GPR142, GPR120, and GPR40 are representatives of GPCRs, which are considered important targets in T2DM therapy. These receptors are often called potential druggable GPCRs. GPR119 is expressed mainly in pancreatic β-cells and in the endocrine cells of the gastrointestinal tract, and it stimulates insulin secretion. For more than 10 years, this target has been very popular in the development of antidiabetic agents. To date, more than a dozen highly effective agents have been developed, a number of which have already been recommended for use in medical practice and are undergoing clinical trials (Figure 16) [71,72].

**Figure 17:**
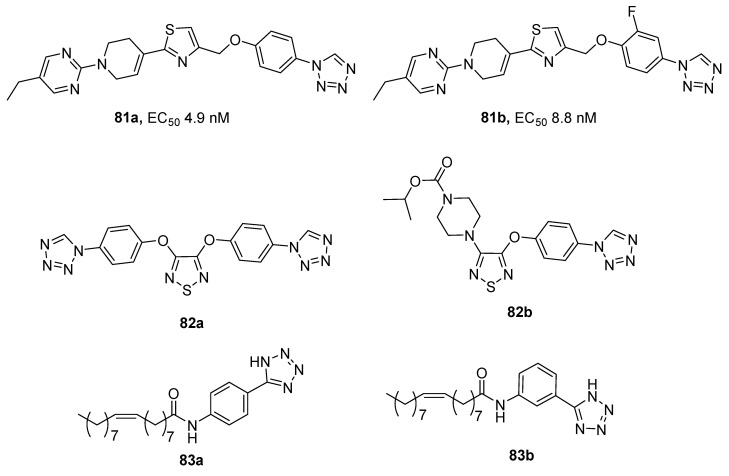
Tetrazole derivatives as promising GPR119 agonists.

As can be seen from Figure 16, low-molecular GPR119 ligands usually contain polynitrogen heterocyclic fragments (triazole, oxadiazole, and tetrazole) linked to other aromatic cycles. These fragments effectively participate in the interaction with the active sites of GPR119, which was confirmed by experimental studies on human GPR119 and theoretical calculations on the structure of ligand-receptor complexes for **79**,**80** [73]. Compound **80**, developed by Arena Pharmaceuticals, was comprehensively studied in vitro and in vivo assays in numerous studies and was approved for phase II clinical trials [71,72,73,74]. However, it has also not been updated for the last clinical status and has not been launched in the drug market. Based on systematic optimization of the general structure MBX-2982, novel terazole-tetrahydropyridine derivatives as novel human GPR119 agonists **81a**,**b** were obtained by Zuo et al. (Figure 17). They showed high GPR119 agonist activity in vitro {**81a** (EC_50_ 4.9 nM) and **81b** (EC_50_ 8.8 nM)}, and in vivo compound **81a** showed a hypoglycemic effect and may have an effect on improving basal metabolic rate in DIO mice [75].

Mankind Pharma collaborators have proposed a series of tetrazole-containing compounds that may act as candidates for GPR119 agonists [76]. These compounds, for example, **82a**,**b**, simultaneously contain 1,2,5-thiadiazole and tetrazole heterocycles. Another type of compound demonstrated that the synthetic derivatives of oleoyl-LPI **83a**,**b** showed promising GLP-1 secreting capabilities in vivo in an animal model of diabetes. Oleoyl-LPI and its derivatives have been shown to induce the release of GLP-1. Moreover, **83a** and **83b** stimulate the cAMP signaling cascade via GPR119. Oleoyl-LPI and **83a** interact with the GPR119 orthosteric site. Compound 83a enhances the acute glucose-stimulated GLP-1 secretion in db/db mice. Based on the results of the biological evaluation and molecular modeling, analogies **83a** and **83b** were hypothesized to be the most potent ligands of GPR119, similar to oleoyl-LPI (Figure 17) [77].

**Figure 18:**
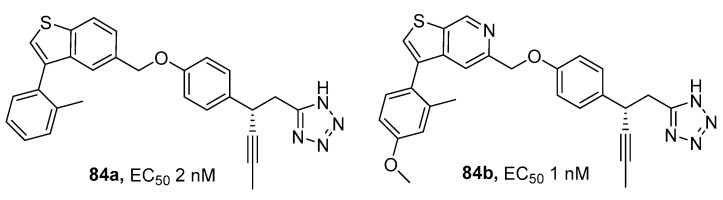
Tetrazole derivatives as promising GPR40 agonists.

Most recently, the recognition of GPR40 as a receptor whose activation enhances glucose-dependent insulin secretion has led to the search for selective agonists for this putative therapeutic target. GPR40, also known as free fatty acid receptor 1 (FFR1), can be endogenously activated by medium-chain saturated and unsaturated fatty acids. The present invention of Janssen Pharmaceutica NV relates to novel compounds **84a**,**b**, which are very active GPR40 agonists and are useful for the treatment of disorders that are affected by the modulation of the GPR40 receptor (Figure 18) [78,79,80]. Compounds were tested in a calcium flux assay using transfected HEK293 cells stably expressing either human GPR40 or male SD rats.

### 3.6. Glycogen Phosphorylases (GP) Inhibitors

**Figure 19:**
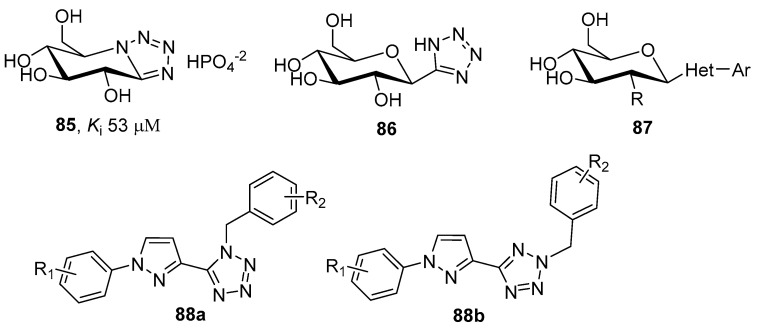
Potential GP inhibitors.

The liver is able to store glucose in the form of glycogen and, through a reverse process, produce and release glucose. The liver is one of the main sources of glucose in the blood and in type 2 diabetes; hepatic glucose production is increased, which can lead to hyperglycemia [81]. Glycogenolysis (the breakdown of glycogen) may account for more than 70% of the hepatic glucose production. The main regulatory enzymes of this system are glycogen phosphorylases (GP), which release glucose 1-phosphate from glycogen. GPa is the phosphorylated isoform, and GPb is the unphosphorylated isoform, modifying its quaternary homodimeric structure. The inhibition of hepatic GP, the main regulatory enzyme in the liver, might be considered a useful target for the control of blood glucose levels. The structure of these inhibitors often contains functional glucose derivatives or related compounds combined with the nitrogen heterocycle. Thus, in 1996, Mithchell et al. investigated condensed nojirimycin tetrazole **85**; it is a competitive inhibitor with respect to glucose 1-phosphate and an uncompetitive inhibitor with respect to phosphate (Figure 19). Tetrazole **85** has poor affinity for GP, but that phosphate substantially improves its binding (*K*_i_(GPb/phosphate) 53 µM) [82]. The structure of the complex (GPb)-**85**-phosphate was demonstrated using calculations and X-ray analysis [83]. Somsák et al., in a series of publications, noted only low activity of 5-(β-D-glucopyranosyl)-1*H*-tetrazole **86**, while some similarly structured triazole, oxadiazole, and imidazole analogs **87** were found to be active at nanomolar concentrations (Figure 19) [84,85,86,87,88]. An in vivo study of isomeric 5-(1-aryl-1*H*-pyrazol-3-yl)-1*H*- and 2*H*-tetrazoles **88a**,**b** as GP inhibitors showed a maximum fall in the blood glucose levels in streptozotocin-induced diabetic Wistar rats, and a molecular docking study has been performed for the potent molecules with GP as target enzyme corroborated the experimental results [89].

### 3.7. α-Glycosidase (AG) Inhibitors

α-Glucosidase is one of the carbohydrate hydrolyzing enzymes that catalyzes the liberation of α-glucose by cleaving the glycosidic bond from the non-reducing portion of the oligomeric substrate. α-Glucosidase inhibitors (AGIs) are oral antidiabetic drugs, preferably used in treating type 2 diabetes mellitus, that delay the absorption of carbohydrates from the gastrointestinal system.

**Figure 20:**
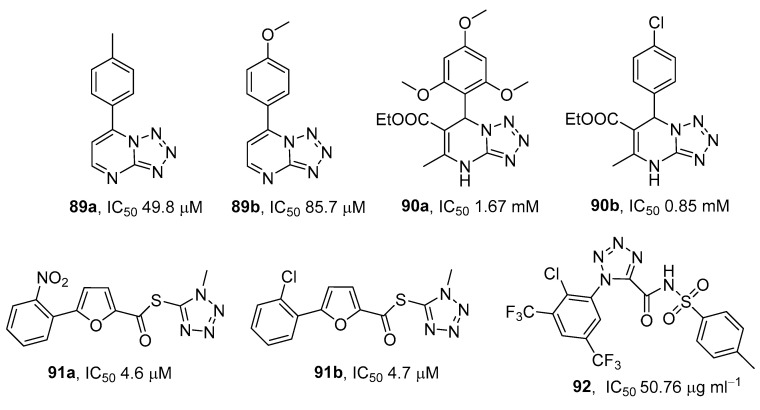
Potential AG inhibitors.

A series of fused tetrazolo[1,5-a]pyrimidine **89a**,**b** derivatives have been synthesized, and their in vitro inhibitory activity for α-glucosidase has been screened using yeast maltase (MAL12) as a model enzyme [90]. The IC_50_ values for most active compounds **89a**,**b** (Figure 20) consist of 49.8 μM and 85.7 μM, respectively, which is comparable with acarbose (33.9 μM). Also, docking studies of the compounds with the active site of the α-glucosidase and in silico prediction of their ADMET (absorption, distribution, metabolism, excretion, toxicity) properties were assessed.

A series of closely related dihydrotetrazolopyrimidines **90a**,**b** were also designed as AG inhibitors [91]. These compounds exhibited better inhibitory activity against α-glucosidase (against maltase and sucrose) than referenced quercetin. On the basis of the work [92], significantly greater inhibitory activity against α-glucosidase was screened for differences in structure 1*H*-tetrazol-5-yl-2,5-disubstituted furanes **91a**,**b**. According to the kinetic study, the most promising compound, **91a** (IC_50_ 4.6 μM), is an uncompetitive inhibitor against α-glucosidase. Molecular docking studies indicated that the existence of the azole group played a critically important role in hydrogen bond interaction with α-glucosidase. Toxicity towards HEK293, RAW264.7, and HepG2 cells suggested that compound **91a** possessed non-toxicity, and it is a good candidate for further investigations. In vitro and in vivo antidiabetic activities of a series of 1-aryl-N-tosyl-1*H*-tetrazole-5-carboxamides were studied [93]. There, compound **92** demonstrated in vitro minimum IC_50_ values against AG and α-amylase (50.76 and 59.38 μg ml^−1^, respectively). α-Amylase catalyzes the cleavage of α-1,4-glycosidic bonds of starch and other related polysaccharides to generate oligosaccharides. Also, tetrazole **92** exhibited a maximum glucose-lowering effect in diabetic rats.

### 3.8. Sodium Glucose Co-Transporter (SGLT) Inhibitors

Sodium-dependent glucose cotransporters (SGLT) are a family of glucose transporters found in the intestinal mucosa of the small intestine (SGLT1) and the renal proximal tubules (SGLT2). SGLT2 protein is the main sodium-dependent glucose transporter, and when SGLT2 protein is inhibited, glucose reabsorption in renal tubules decreases, which leads to glucose excretion in the urine with a subsequent decrease in plasma glucose levels [49]. At the same time, SGLT inhibition can suppress secondary diseases caused by T2DM.

**Figure 21:**
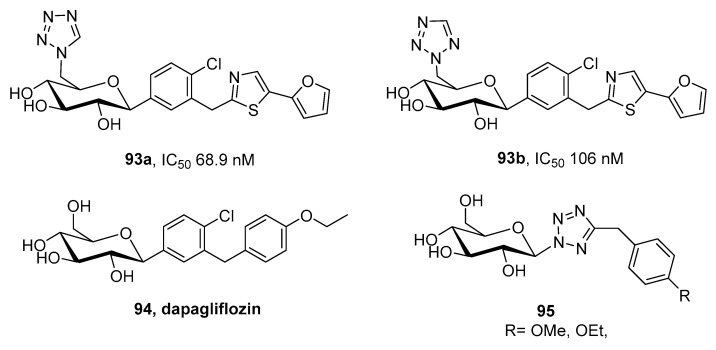
SGLT2 inhibitors.

A synthesis and antidiabetic activity (in vitro and in vivo) of a wide series of novel tetrazole-bearing glycosides have been described in several papers and patents (Figure 21) [94,95,96,97]. Thus, directly bonded 1*H*- and 2*H*-tetrazoles **93a**,**b** showed only moderate activity in vitro (IC_50_ 68.9 and 106 nM) in comparison with control selective SGLT2 inhibitor dapagliflozin **94** (EC_50_ 1.1 nM) [95]. Moreover, 5-Arylmethyltetrazoles bearing glycosides were examined in vivo by means of a mouse oral glucose tolerance test [94]. The D-glucose derivatives were found to be more potent inhibitors than their D-galactose counterparts, and usually, the 2-glucosyisomer had higher activity than 1-glucosyltetrazoles. Among these compounds, N-glucosyltetrazoles **95** had better activity in blood glucose level by oral glucose tolerance (R=OEt, 77.0%; R=Ome, 73.9%) compared to reference dapagliflozin **94** (68%).

### 3.9. Other Types of Activities

In this section, we have discussed those activities that are less discussed above in the context of antidiabetic agents containing tetrazolyl moiety, and the results of such studies are presented in one or two publications. These include, by the way, very important targets for the action of modern antidiabetic agents, which are intensively studied today [49].

**Figure 22:**
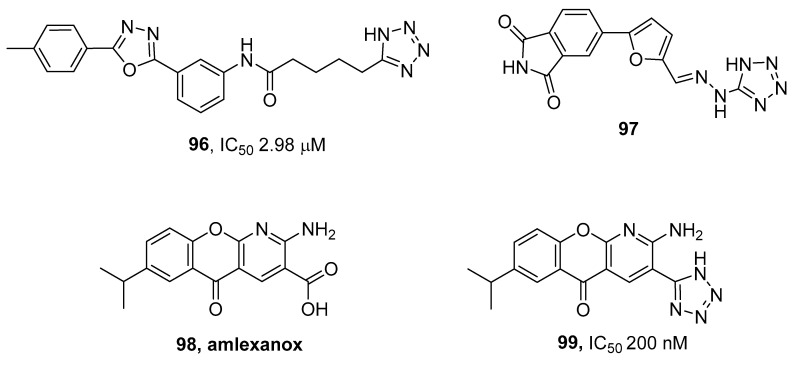
Different types of antidiabetic agents: fructose-1,6-bisphosphatase (FBPase) inhibitor **96**, prolyl hydroxylase 2 inhibitor (PHD2) **97**, IkB kinase ε (IKKε), and TANK binding kinase 1 (TBK1) inhibitors **98**, **99**.

Here, first of all, the drug design of fructose-1,6-bisphosphatase (FBPase) inhibitors should be mentioned. FBPase, more precisely the liver form of FBPase (FBP1), is a key enzyme of gluconeogenesis in both the liver and kidneys, which contributes greatly to the development of hyperglycemia in T2DM patients. This target is also of extensive interest because drug therapy does not lead to hypoglycemia, which may be associated with the effects of other antidiabetic agents. A series of 2,5-diphenyl-1,3,4-oxadiazole derivatives were synthesized, and compound **96**, bearing a terminal 1*H*-tetrazole moiety, demonstrated remarkable inhibition of gluconeogenesis by the inhibition of FBPase in vitro (IC_50_ 2.98 µM) (Figure 22) [98]. In addition, this compound demonstrated an inhibition of glucose output in rat hepatocytes and excellent membrane permeability.

Dysfunctions of hypoxia-inducible factor (HIF-1α) in diabetes have been indicated to deteriorate wound closure, whereas stabilization of HIF-1α has been shown to improve wound healing and osteoarthritis. Prolyl hydroxylase 2 (PHD2) is a negative regulator of HIF-1α, and a deficiency in PHD2 stabilized HIF-1α. Therefore, PHD2 inhibitors are tools for the treatment of post-diabetic disorders. Based on a virtual screening, compound **97** was identified as a potential PHD2 inhibitor (Figure 22) [99]. It was also shown that tetrazole **97** promotes the migration and capillary tube formation capacity of human umbilical vein endothelial cells through enhancing the stability of HIF-1α.

Obesity is a leading risk factor for the development of type 2 diabetes and is accompanied by inflammation of the liver and adipose tissue, leading to impaired metabolism. This leads to increased expression of IkB kinase ε (IKKε) and TANK binding kinase 1 (TBK1), which contributes to additional weight gain and further decreased insulin sensitivity. Amlexanox **98** inhibits IKKε and TBK1 to improve insulin sensitivity (IC_50_ 5800 and 800 nM). Analog **99**, which contained a tetrazole moiety in place of the carboxylic acid, displayed improved potencies: IC_50_ 400 and 200 nM against IKKε and TBK1, respectively [100]. A treatment activity on the diabetic retinopathy of Cu(II)-containing coordination polymer with 5-[3-(1*H*-imidazol-1-yl)phenyl]-1*H*-tetrazolate bifunctional ligand was studied, and the related mechanism was discussed [101].

Inhibition of 11β-hydroxysteroid dehydrogenase type 1 (11β-HSD1) is another area for the development of new antidiabetic agents that has received considerable attention [49]. This enzyme is mainly found in the liver, adipose tissue, and CNS, where it catalyzes the conversion of physiologically inactive cortisone to the active hormone cortisol. The latter activates the risk of metabolic syndrome, insulin resistance, glucose tolerance, hypertension, and other manifestations of T2DM. Some tetrazoles, discussed above as agents against other targets, exhibit such activity. Thus, compound **50**, according to studies on the cellular line HEK293, is an inhibitor of 11β-HSD1 to a greater extent than clofibrate **26** [46,47]. Also, tetrazolyl ureas and biguanides were tested in vivo on glucocorticoid-induced insulin resistance models, and the most active tetrazolo[1,5-c]quinazolin-5(6*H*)-one **48** (R=H) exceeded the reference drugs metformin **1** and gliclazide.

## 4. Concluding Remarks

The tetrazolyl pharmacophore moiety is widely used in the drug design of novel antidiabetic agents against T2DM and related (associated) diseases. Tetrazole derivatives have been investigated as agents against major biological targets, including the following: peroxisome proliferator-activated receptors (PPARs) agonists, protein tyrosine phosphatase 1B (PTP1B) inhibitors, aldose reductase (AR) inhibitors, dipeptidyl peptidase-4 (DPP-4) inhibitors, glucagon-like peptide 1 (GLP-1) agonists, G protein-coupled receptor (GPCRs) agonists, glycogen phosphorylases (GP) inhibitors, α-glycosidase (AG) inhibitors, sodium glucose co-transporter (SGLT) inhibitors, fructose-1,6-bisphosphatase (FBPase) inhibitors, IkB kinase ε (IKKε) and TANK binding kinase 1 (TBK1) inhibitors, and 11β-hydroxysteroid dehydrogenase type 1 (11β-HSD1). Impressive advances have been made in the development of antidiabetic agents acting as selective PPARγ agonists, with examples of tetrazol-5-yl-1,2,3,4-tetrahydroisoquinolines **38** and 5-[3-[6-(5-methyl-2-phenyl-4-oxazolylmethoxy)-3-pyridyl]propyl]-1*H*-tetrazole **40** (EC_50_ 6–15 nM); aldose reductase (AR) inhibitors on [5-(3-thienyl)tetrazol-1-yl]acetic acid **69** (IC_50_ 24 nM); DPP-4 inhibitors on tetrazolo[1,5-a]pyrimidine **75** (IC_50_ 14 nM); and high active polycyclic tetrazole-containing systems such as GPR119 agonists **80**, **81**, **84** (EC_50_ 3.9–8.8 nM) and GPR40 agonists **84** (EC_50_ 1–2 nM). According to numerous theoretical calculations, the tetrazole heterocycle is actively involved in binding to biological targets. In many cases, the tetrazole-containing leader compounds markedly exceed the activity of medications already known and used in T2DM therapy in vitro and in vivo. Some of them are undergoing clinical trials. In addition, tetrazole derivatives are very often used to act on diabetes-related targets or to treat post-diabetic disorders. Thus, the tetrazole scaffold is promising for further research to develop effective antidiabetic drugs.

## Data Availability

Not applicable.
